# Leptin mediate central obesity on the severity of cardiovascular autonomic neuropathy in well-controlled type 2 diabetes and prediabetes

**DOI:** 10.1186/s12967-020-02559-7

**Published:** 2020-10-19

**Authors:** Yun-Ru Lai, Meng Hsiang Chen, Wei Che Lin, Wen-Chan Chiu, Ben-Chung Cheng, Jung-Fu Chen, Nai-Wen Tsai, Chih-Cheng Huang, Cheng-Hsien Lu

**Affiliations:** 1 Department of Biological Science, National Sun Yat-Sen University, Kaohsiung, Taiwan; 2 Departments of Neurology, Kaohsiung Chang Gung Memorial Hospital, Chang Gung University College of Medicine, No. 123, Ta Pei Road, Niao Sung Hsiang, Kaohsiung City, 833 Taiwan; 3 Departments of Radiology, Kaohsiung Chang Gung Memorial Hospital, Chang Gung University College of Medicine, Kaohsiung, Taiwan; 4 Departments of Internal Medicine, Kaohsiung Chang Gung Memorial Hospital, Chang Gung University College of Medicine, Kaohsiung, Taiwan; 5 Center for Shockwave Medicine and Tissue Engineering, Kaohsiung Chang Gung Memorial Hospital, Chang Gung University College of Medicine, Kaohsiung, Taiwan; 6 Department of Neurology, Xiamen Chang Gung Memorial Hospital, Xiamen, Fujian China

**Keywords:** Cardiovascular autonomic neuropathy, Cardiac autonomic reflex tests, Central obesity, Composite autonomic scoring scale, Leptin, Type 2 diabetes and prediabetes, Waist circumference

## Abstract

**Background:**

Evidences support the view that central obesity is an independently cardiovascular risk. It is thought that leptin contributes to autonomic dysfunction and cardiovascular risks in type 1 and type 2 diabetes mellitus (T1DM and T2DM). This raises the possibility that leptin might mediate the relationship between central obesity and the severity of cardiovascular autonomic neuropathy (CAN) in patients with well-controlled T2DM and prediabetes.

**Methods:**

The complete cardiovascular reflex tests and biomarkers were assessed for each patient. The severity of CAN was assessed using composite autonomic scoring scale (CASS). A single-level three-variable mediation model was used to investigate the possible relationships among central obesity [as indicated by waist circumference (WC)], leptin level, and severity of CAN (as indicated by CASS value).

**Results:**

A total of 107 patients were included in this study: 90 with diabetes and 17 with prediabetes. The results demonstrate that increased WC is associated with increased severity of CAN (r = 0.242, P = 0.017). We further discovered that leptin level is positively correlated with WC (r = 0.504, P < 0.0001) and the CASS value (r = 0.36, P < 0.0001). Further mediation analysis shows that leptin level serves as mediators between higher WC and higher CASS.

**Conclusions:**

Our results highlighted the relationship among leptin, central obesity, and severity of CAN. As the leptin level serves as mediator between central obesity and severity of CAN, a longitudinal study is needed to confirm that control of WC can decrease leptin levels and can be effective in reducing CAN progression.

## Background

The prevalence of obesity has increased worldwide over the past several decades to become a global health problem, and it poses an increased risk of multiple serious conditions [1, 2], including type 2 diabetes (T2DM), cardiovascular diseases, nonalcoholic fatty liver disease, chronic kidney disease, and different types of cancer [3].

Currently, adipose tissue is recognized as a complex and dynamic endocrine organ in the body that not only stores energy, but also regulates energy homeostasis, cellular reactions, and metabolic homeostasis [4]. The adipocytes are metabolically active and potent secretory cells, capable of releasing many adipocytokines, and are involved in the regulation of appetite, inflammatory and immune functions, glucose and lipid metabolism, long-term energy balance, insulin sensitivity of insulin responsive tissues, cardiovascular homeostasis and reproduction, and other important biological and physiological functions [5]. Leptin is an adipocyte-secreted hormone with a key role in energy homeostasis, and it can also stimulate the secretion of several cytokines via inflammatory cells. Additionally, serum leptin levels are positively correlated with insulin resistance (IR) [6].

The pathophysiological mechanism of cardiovascular autonomic neuropathy (CAN) is multifactorial, and there is sufficient evidence that it may precede diabetes mellitus (DM) [7]. MetS is associated with multiple risk factors, including central obesity that may increase the risk of cardiovascular events in individuals with type 2 DM [8]. MetS with central obesity is associated with an imbalance of homeostatic mechanisms, leading to adipose tissue dysfunctionality characterized by altered secretion of adipokines. This condition is also associated with a special upregulation in the expression of pro-inflammatory adipokines [9] and increase in the generation of free radicals and other reactive species, leading to increased oxidative stress, production of cell adhesion molecules, and microcirculation dysfunction [10].

The prevalence of CAN is variable and is dependent on the definition and criteria used for the diagnosis. Toronto Consensus recommends using four cardiovascular reflex tests (CARTs) and frequency-domain tests as a sensitive and specific method in assessing the presence of CAN in patients with DM [11]. Furthermore, the American Academy of Neurology’s summary of evidence-based guidelines for clinicians recommends that a combination of autonomic screening tests and composite autonomic scoring scales (CASS) should be considered to achieve the highest diagnostic accuracy of CAN [12]. These two diagnostic methods and scoring systems are commonly used in research and clinical practice.

CAN has a strong influence on various cardiovascular diseases and leads to severe morbidity and mortality in patients with DM [13, 14]. To our knowledge, only a few studies have demonstrated that leptin is associated with autonomic dysfunctions in type 1 diabetes mellitus (T1DM) and T2DM [2, 15–17]. To date, there is a paucity of information that focuses on the relationship among central obesity, leptin level and severity of CAN in patients withT2DM.

According to our hypotheses, central obesity is associated with upregulation in the expression of leptin, and leptin might affect autonomic function by acting both centrally and peripherally and thus leads to a decline in CAN. Our results may be beneficial for the development of therapeutic strategies for patients with DM and may help improve the quality of life of patients with T2DM and prediabetes.

## Patients and methods

### Study population

A total of 107 patients (≥ 20 years of age) who visited the outpatient diabetic clinic at Kaohsiung Chang Gung Memorial Hospital (CGMH) in Taiwan were included in this study: 90 subjects with type 2 diabetes and 17 subjects with prediabetes. Exclusion criteria were moderate-to-severe heart failure (NYHA class III and IV), presence of any type of arrhythmia that prevents analysis of heart rate variability (HRV), or pacemaker implantation. This study was approved by the Ethics Committee of Chang Gung Memorial Hospital Institutional Review Board (201800388B0C501 and 201901363B0).

### Baseline clinical measurements

All patients underwent complete neurological and physical examinations on enrollment and on follow-up visits at the outpatient clinic. A detailed medical history regarding prior use of medications was obtained from every patient and their families through standardized questions. Demographic data, including age, sex, duration of diabetes (years), body mass index (BMI), systolic and diastolic blood pressure (SBP and DSP, respectively), waist circumference (WC) during autonomic function testing, underlying diseases (hypertension, coronary artery disease, ischemic stroke, and diabetic retinopathy [DR]), and laboratory parameters, were obtained at baseline. MetS was evaluated according to the updated National Cholesterol Education Program/Adult Treatment Panel III criteria [18]. A subject who had at least three of the following components was defined as having MetS: (1) central obesity: WC ≥ 90 cm for men and ≥ 80 cm for women; (2) hypertension: SBP ≥ 130 mmHg or DBP ≥ 85 mmHg or drug treatment for hypertension; (3) fasting blood glucose ≥ 100 mg/dL or diagnosed diabetes; (4) abnormal high-density lipoprotein (HDL) level: HDL cholesterol level < 40 mg/dL for men and < 50 mg/dL for women or drug treatment for low HDL cholesterol (HDL-C); and (5) abnormal triglyceride (TG) level: TG level ≥ 150 mg/dL or drug treatment for high TGs. The cut-off values for obesity, based on BMI, should be much lower in Taiwan than in Western countries. We determined that the optimal cut-off values for our study were BMIs of 23.6 in men and 22.1 in women, and WCs of 80.5 cm in men and 71.5 cm in women may be more appropriate to define adult overweight or obesity in Taiwan [19].

### Laboratory measurements

Blood samples were obtained by antecubital vein puncture in a fasting, non-sedative state between 09:00 and 10:00 a.m in the control and study groups to exclude the possible influence of circadian variations. All blood samples were collected into Vacutainer SST tubes (BD, Franklin Lakes, NJ) and centrifuged at 3000 rpm for 10 min; subsequently, serum samples were collected and stored at − 80 °C in multiple aliquots, prior to biochemical measurement. Serum levels of TGs, total cholesterol, HDL-C, low-density lipoprotein cholesterol, blood sugar, glycohemoglobin (HBA1c), and high-sensitive C-reactive protein (hs-CRP) were analyzed by the hospital’s central laboratory. The homeostasis model assessment of insulin resistance (HOMA-IR index) was calculated by fasting glucose (in mmol/L) × fasting insulin (in mU/ml)/22.5. In our study, we defined IR as ≥ 2, based on the Taiwanese population [20]. Additionally, we used the TG/HDL ratio as a surrogate marker for IR [20].

The estimated glomerular filtration rate (eGFR) in each patient was calculated using an equation for Chinese subjects, as previously described [21]. The normal rate of albumin excretion is less than 30 mg/day; therefore, persistent albumin excretion between 30 and 300 mg/day is classified as microalbuminuria and albumin excretion above 300 mg/day is considered macroalbuminuria [22].

### Biomarkers for oxidative stress

We evaluated the oxidative stress condition in all subjects by measuring the serum thiobarbituric acid-reactive substance (TBARS) and thiol levels. Serum TBARS levels were measured using a well-established method for detecting lipid peroxidation with a commercially available assay kit (Cayman Chemical, Ann Arbor, MI, USA, cat. no. 10009055). The Assay Kit was used according to the manufacturer’s instructions, as previously described [23]. The values for the samples were calculated using a linear calibration curve, which was prepared using pure malondialdehyde-containing samples (range, 0–50 μmol/L).

The ability of anti-oxidative defense in response to increased oxidative damage was evaluated by measuring the serum levels of total reduced thiols because serum thiols are physiologic free radical scavengers. Serum total protein thiols were estimated by directly reacting thiols with 5,5-dithiobis 2-nitrobenzoic acid to form 5-thio-2-nitrobenzoic acid (TNB). The number of thiols in the sample was calculated based on absorbance, which was determined using the extinction coefficient of TNB (A412 = 13,600/M/cm).

### ELISA Analysis for biomarkers of endothelial dysfunction and leptin

Serum sICAM-1 and sVCAM-1 levels were assessed using commercially available ELISA kits (R&D Systems, Minneapolis, USA; sICAM-1: cat. no. DCD 540; sVCAM-1: cat.no. DVC00), as previously described [24]. Leptin and adiponectin levels were evaluated by enzyme-linked immunosorbent assay in the quality-controlled central laboratory of the CGMH.

### Assessment of cardiovascular autonomic functions

All subjects underwent a standardized evaluation of cardiovascular autonomic function. CARTs are considered gold-standard measures of autonomic function in patients with DM [11]. Parameters, which were computed as Ewing’s methods, including heart rate responses to deep breathing (E:I ratio), to standing (30:15 ratio), and to the Valsalva maneuver and blood pressure responses to standing [25], were often used by diabetologists. CAN was defined with the presence of at least two abnormal test results [11].

### Scoring of severity in cardiovascular autonomic neuropathy

The severity of CAN in our study was assessed using the cardiovagal and adrenergic sub-scores of the CASS [26]. The test battery comprised heart rate response to deep breathing (HR_DB), Valsalva ratio (VR), and 5-min head-up tilt (HUT) tests, as described by Low [27]. The detailed methodology for computing HR_DB and VR were based on a previous study [27]. The CASS had a scale from 0 to 7 points in this study [28].

### Statistical analysis

**Fig. 1:**
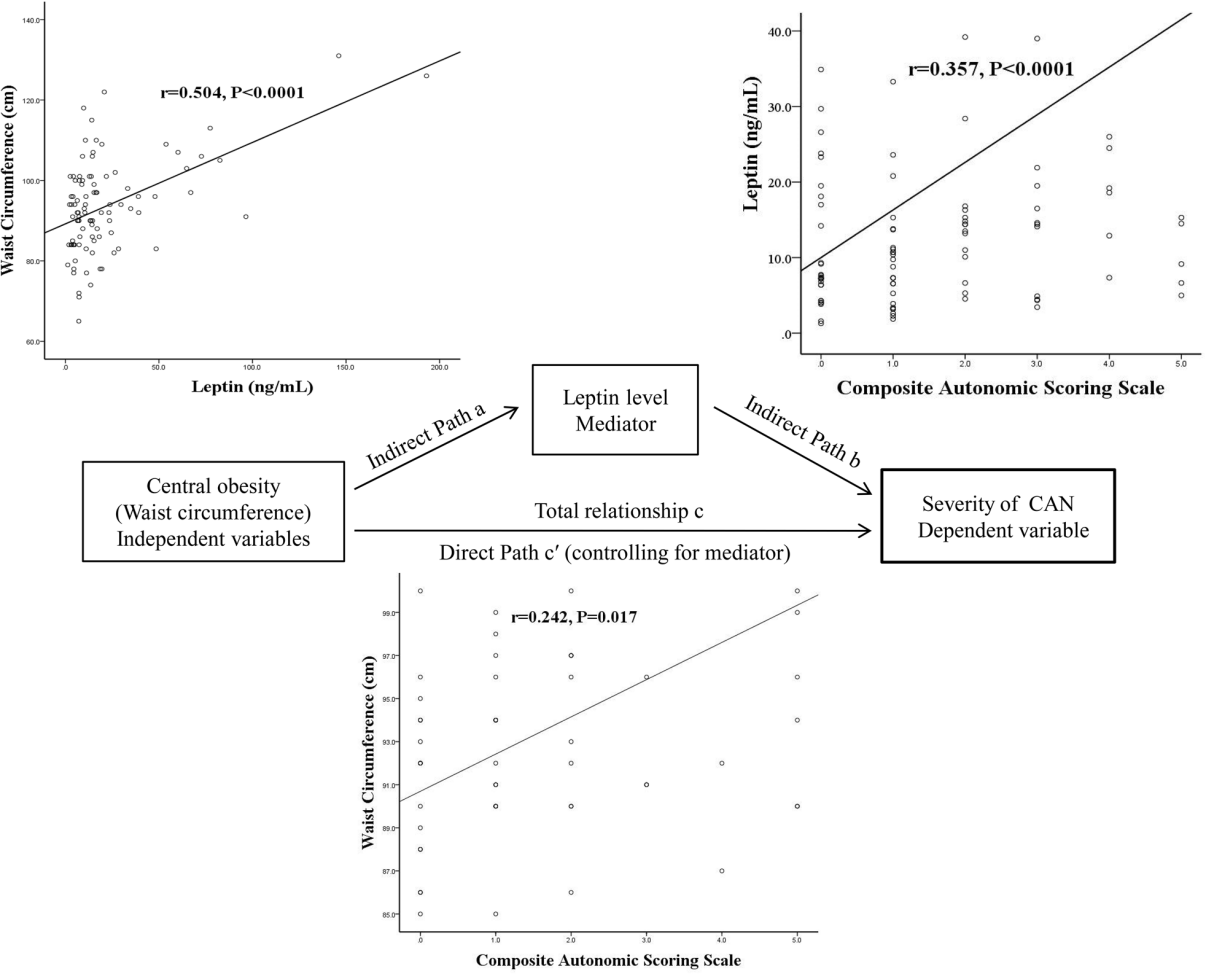
The diagram of the mediation hypothesis framework

Data are expressed as means ± standard derivations or medians (interquartile ranges). Categorical variables were compared using Chi-square or Fisher’s exact tests. Continuous variables that were not normally distributed by Kolmogorov–Smirnov test were logarithmically transformed to improve normality and compared. Three separate statistical analyses were performed. First, patients were stratified into two groups (diabetes and prediabetes, and presence or absence of CAN) and compared. Second, correlation analysis was used to evaluate the relationship between the leptin and variables that included age, diabetes duration, BMI, WC, SPB, and DSP, peripheral blood studies for vascular risks and autonomic parameters. Finally, a single-level three-variable mediation model [29], illustrated in Fig. 1, was used to investigate the causal relationships between leptin (mediating variable), central obesity (wrist circumference, independent variable), and severity of CAN (CASS, dependent variable). Mediation analysis tests whether the direct effect of an independent variable on a dependent variable can be explained by the indirect influence of the mediating variable. A significant mediator is one whose inclusion as an intermediate variable significantly affects the relationship between the independent and dependent variables. The statistical significance threshold in Sobel test was set at 0.05 for all the relevant paths [30]. All statistical analyses were conducted using the SAS software package, version 9.1 (2002, SAS Statistical Institute, Cary, North Carolina).

## Results

### General characteristics of the patients with diabetes and prediabetes

A total of 107 patients were included in this study: 90 with diabetes and 17 with prediabetes. Patient characteristics and baseline underlying diseases at assessment are presented in Table 1. The mean diabetes duration in the diabetes group was 11.5 ± 8.6 years. The significant differences between the diabetes and prediabetes groups included HDL-C (mmol/L) (P = 0.017), HbA1c (%) (P < 0.0001), eGFR (P = 0.002), and UACR (P < 0.0001), BMI (p = 0.001), WC (p = 0.001), presence of chronic kidney diseases (p = 0.002), and proteinuria (p = 0.001).

**Table 1:** Baseline characteristics of patients with Type 2 diabetes and prediabetes

	Prediabetes (n = 17)	Diabetes (n = 90)	P-value
*Characteristics*			
Age (year)	63.3 ± 12.8	67.3 ± 8.1	0.21
Sex (male/female)	11/6	50/40	0.49
Diabetes duration (year)	–	11.5 ± 8.6	
Body mass index	24.3 ± 2.5	27.1 ± 4.6	0.001*
Waist circumference (cm)	85.3 ± 7.5	94.6 ± 11.7	0.001*
SBP (mmHg)	131.6 ± 14.8	137.8 ± 17.9	0.07
DBP (mmHg)	76.9 ± 8.2	76.5 ± 12.5	0.90
*Baseline underlying disease*			
Hypertension (%)	11 (64.7%)	68 (75.6%)	0.38
Coronary heart disease (%)	2 (11.8%)	11 (12.2%)	1.0
Cerebrovascular events (%)	4 (23.5%)	33 (36.7%)	0.30
Hyperlipidemia (%)	14 (82.4%)	71 (78.8%)	1.0
Chronic kidney diseases (%)	2 (11.8%)	47 (52.2%)	0.002*
Retinopathy, n (%)	5 (29.4%)	71 (78.9%)	0.24
Proteinuria, n (%)	0	37 (41.1%)	0.001*
*Laboratory test findings*			
Total cholesterol(mmol/L)	169.6 ± 27.5	156.4 ± 34.2	0.135
Triglyceride(mmol/L)	119.4 ± 70.7	140.7 ± 75.6	0.286
HDL-C (mmol/L)	53.2 ± 11.2	45.6 ± 11.2	0.017*
LDL-C (mmol L)	91.8 ± 21.1	85.3 ± 26.7	0.348
HbA1c (%)	6.0 ± 0.3	7.1 ± 0.8	< 0.0001**
eGFR	78.1 ± 25.8	57.3 ± 25.3	0.002*
UACR	5.8 (3.6, 7.2)	17.3 (8.5, 85.3)	< 0.0001**
TG/HDL ratio	2.5 ± 1.8	3.4 ± 2.1	0.11
HOMA-IR	2.4 ± 2.3	3.5 ± 2.7	0.135
hs-CRP	0.6 (0.3, 2.7)	0.9 (0.6, 2.0)	0.336
*Type of diabetes treatment*			
OHA only (%)	0	74 (82.2%)	
Insulin only (%)	0	4 (4.4%)	
Insulin + OHA (%)	0	7 (7.8%)	
Diet control without medication (%)	17 (100%)	5 (5.6%)	
*Other concomitant medications*			
ACE inhibitor or ARB (%)	11 (64.7%)	65(72.2%)	
Beta-blocker (%)	3(17.6%)	28 (31.1%)	
Calcium channel blocker (%)	4 (23.5%)	45 (50%)	
Diuretics (%)	3 (17.6%)	18 (20%)	
Antiplatelet medications (%)	4 (23.5%)	26 (28.9%)	
Statins (%)	13 (76.4%)	70 (77.8%)	

Data are presented as means ± standard deviations or median (IQR)

*n* number of cases, *IQR* interquartile range, *SBP* systolic blood pressure, *DBP* diastolic blood pressure, *HDL-C* high-density lipoprotein cholesterol, *LDL-C* low-density lipoprotein cholesterol, *HbA1c* glycohemoglobin, *eGFR* estimated glomerular filtration rate, *hs-CRP* high-sensitive C-reactive protein, *UACR* urine albumin-creatine ratio, *HOMA-IR* homeostasis model assessment of insulin resistance, *TG/HDL* Triglyceride/LDL-C, *OHA* oral hypoglycemic agent, *ACE* angiotensin-converting enzyme, *ARB* angiotensin II receptor blocker

^*^ indicates that p value < 0.05.

** indicates that p value < 0.0001

### Parameters of cardiovascular autonomic study between patients with and without CAN

Patients with CAN had higher CASS values and lower levels of parasympathetic parameters, which include HR_DB (beats/min), VR, E:I ratio, 30:15 ratio, and sympathetic parameters, such as blood pressure responses to standing (Tables 2, 3).

**Table 2:** Baseline cardiovascular autonomic study with and without cardiovascular autonomic neuropathy

	No CAN (n = 59)	CAN (n = 48)	P-value^a^
CASS	0.5 ± 0.4	3.2 ± 1.1	< 0.0001***
HR_DB (beats/min)	11.1 ± 7.0	5.5 ± 3.9	< 0.0001***
Valsalva ratio	1.4 ± 0.2	1.2 ± 0.1	< 0.0001***
Delta SBP	-3.0 (-10.0, 2.0)	-8 (-17.0, -2.0)	0.004**
E:I ratio	1.2 ± 0.1	1.1 ± 0.04	< 0.0001***
30/15 ratio	1.1 ± 0.1	1.0 ± 0.03	< 0.0001***

Data are presented as means ± standard deviations or median (IQR); n (%).

*n* number of cases, *IQR* interquartile range, *CASS* Composite Autonomic Scoring Scale, *HR_DB* heart rate response to deep breathing, *CAN* cardiac autonomic neuropathy, *Delta SBP* The change between minimum systolic blood pressure during head-up tilt and baseline systolic blood pressure

^a^Independent t-test, two-side, data were logarithmically transformed to improve normality

*P < 0.05

**P < 0.01

*** = P < 0.001

**Table 3:** Correlation analysis between leptin levels and parameters of biomarkers and cardiovascular autonomic functions

Variables	Leptin, ng/mL	
	r	P value
Age (year)	−0.10	0.33
Diabetes duration (year)	−0.023	0.821
Body mass index	0.588	< 0.0001**
Waist circumference (cm)	0.504	< 0.0001**
SBP (mmHg)	0.007	0.946
DBP (mmHg)	−0.053	0.597
HbA1c (%)	0.175	0.076
eGFR	−0.175	0.09
UACR	0.351	0.001**
hs-CRP	0.291	0.006**
TBARS, μmol/L	0.259	0.008**
Thiols, μmol/L	−0.25	0.011*
sICAM-1 (ng/ml)	0.122	0.216
sVCAM-1 (ng/ml)	0.109	0.271
Triglyceride/HDL-C ratio	0.346	< 0.0001
HOMA-IR	0.304	0.008
HR_DB	−0.30	0.002**
Valsalva ratio	−0.31	0.002**
Delta SBP	0.031	0.80
E:I ratio	−0.26	0.008**
30/15 ratio	−0.18	0.07

*n* number of cases, *SBP* systolic blood pressure, *DBP* diastolic blood pressure, *HDL-C* high-density lipoprotein cholesterol, *LDL-C* low-density lipoprotein cholesterol, *HbA1c* glycohemoglobin, *eGFR* estimated glomerular filtration rate, *hs-CRP* high-sensitive C-reactive protein, *UACR* urine albumin-creatine ratio, *HOMA-IR* homeostasis model assessment of IR, *HR_DB* heart rate response to deep breathing, *Delta SBP* The change between minimum systolic blood pressure during head-up tilt and baseline systolic blood pressure

^*^P < 0.05

**P < 0.01

***P < 0.001

### Correlation analysis between leptin levels and parameters of biomarkers and cardiovascular autonomic functions

Correlation analysis parameters that are used to test the relationship between leptin level and parameters of biomarkers and cardiovascular autonomic functions are listed in Table 4. The significant statistical results (correlation coefficient, P-value) between leptin level and parameters of biomarkers were as follows: BMI (r = 0.588, P < 0.0001), WC (r = 0.504, P < 0.0001), UACR (r = 0.351, P = 0.001), hs-CRP (r = 0.291, P = 0.006), TBARS (r = 0.259, P = 0.008), thiols (r = -0.25, P = 0.011), TG/HDL-C ratio (r = 0.346, P < 0.0001), and HOMA-IR (r = 0.304, P = 0.008). Further, significant statistical results (correlation coefficient, P-value) between leptin levels and parameters of cardiovascular autonomic functions were as follows: HR_DB (r = -0.30, P = 0.002), Valsalva ratio (r = −0.31, P = 0.002), E:I ratio (r = −0.26, P = 0.008), and 30/15 ratio (r = −0.18, P = 0.07) (Table 3).

**Table 4:** A simple mediation model of central obesity (X) on severity of cardiovascular autonomic neuropathy (ϒ) through leptin effort (M)

	Path coefficient	Standard error	P-value
*Total effects (total relationship, path c) *^*Ω*^			
The relationship between the independent and dependent variables	0.033	0.236	0.022
*Direct effects, path c′*			
The relationship between the independent and dependent variables by including the mediator into the model	0.01	0.074	0.058
*Indirect effect, path a*			
The effect of the independent variable on the mediator	1.252	0.22	< 0.0001
*Indirect effect, path b*			
The effect of the mediator on the dependent variable by controlling the effect for the independent variables	0.02	0.005	< 0.0001

*X* independent variable (central obesity: waist circumference), *ϒ* dependent variable (severity of cardiovascular autonomic neuropathy: composite autonomic scoring scales, *M* mediator (leptin level), *Ω* The mediation effects a × b which is defined as the reduction of the relationship between the independent and dependent variables (central obesity-severity of cardiovascular autonomic neuropathy) (total relationship, path c) by including the mediator into the model (direct path, path c′), axb = 1.252 × 0.02 = 0.0245 = c–c′ (sobel test, P = 0.011)

^*^indicates that p value < 0.05

**indicates that p value < 0.0001

### Mediation analysis for central obesity, severity of cardiovascular autonomic neuropathy and leptin level

The primary hypothesis of this analysis concerns whether the effect of central obesity (WC, independent variable) on the severity of CAN (CASS, dependent variable) was explained indirectly by leptin level (mediator) with significant group main effect. The path model jointly tested three effects of interest that are required if leptin level links WC with the CASS: (a) the effect of the independent variable (WC value) on the mediator (leptin level) (indirect effect, path a); (b) the effect of the mediator on the dependent variable (CASS) by controlling the effect for the leptin level (indirect effect, path b); and (c) the mediation effect a × b, which is defined as the reduction of the relationship between the independent and dependent variables (WC and CASS) (total relationship, path c) by including the mediator into the model (direct path, path c′). For simplicity, we report a full list of the results from the present study that fulfill the three criteria cited previously. The mediation relationship was significant (*p* = 0.011 in Sobel test) (Fig. 1) (Tables 4).

## Discussion

### Major findings of our study

Consistent with our hypothesis and in line with the extant literature, patients with T2DM and prediabetes experienced higher WC, higher leptin level and worse cardiovascular autonomic function. All vascular risk factors, including blood pressure, blood glucose, and lipid profiles, were well-controlled, except for central obesity, a modified risk factor, which was not corrected in our study. Our study also showed that leptin level is significantly correlated with BMI and WC. Thus, our results highlighted the relationship among leptin, central obesity, and severity of CAN.

### Pathogenesis of leptin and inflammation, oxidative stress, and endothelial dysfunction

Visceral adipose tissue is a bioactive organ that secretes several adipokines, and is a source of proinflammatory and proatherogenic cytokines [31]. The rapid expansion of adipocyte size in obese individuals occurs in an uncoordinated manner, which leads to altered secretion of adipocytokines with a special upregulation in the expression of pro-inflammatory adipocytokines [9], as well as impaired angiogenesis, endothelial dysfunction, and microvascular complications [10].

Leptin and its receptors structurally resemble proinflammatory cytokines and their receptors [32]. In addition, proinflammatory mediators increase leptin secretion, and leptin levels increase considerably during inflammation [33]. Leptin may also increase oxidative stress via activation of the Rho and Rac family of small GTPase [34, 35]. Due to the proinflammatory and prooxidant properties of leptin, hyperleptinemia produces systemic endothelial dysfunction [34]. Our study results showed a positive association between leptin levels and inflammation (hs-CRP), increased oxidative stress (TBARs), and decreased antioxidative capacity (thiol). It also showed that leptin levels are positively associated with biomarkers of microvascular complications of diabetes (e.g., diabetic kidney diseases and CAN).

### Association among leptin, obesity, IR, and cardiac autonomic function in diabetes and pre-diabetes

Leptin is a hormone predominantly synthesized by adipocytes and enterocytes in the small intestine that helps regulate energy balance and acts on cell receptors in the arcuate nucleus of the hypothalamus [6]. Although the regulation of fat stores is deemed to be the primary function of leptin, it also plays a role in other physiological processes. Leptin levels are generally elevated in obesity, and obese individuals are often leptin resistant, as seen in the failure of recombinant leptin to cause weight loss. Hyperleptinemia shunts excess free fatty acids ectopically to non-adipose tissue and diverts fatty acids in those tissue to storage rather than to oxidative consumption [36]. The lipotoxicity, ensuing from this ectopic accumulation of intracellular TGs, contributes to the dysfunction of these organs, and is a critical determinant of IR [37].

Although leptin levels are closely associated with adiposity and increased MetS components, the role of leptin levels in diabetes and prediabetes groups is rather controversial [38, 39]. A recent study from Japan shows that plasma leptin level is comparable between diabetic and non-diabetic patients, despite the fact that BMI, visceral fat area, and subcutaneous fat area are significantly higher in patients with diabetes [15]. Another study in relatively lean rural Chinese adults found that plasma leptin levels are associated with IR and prediabetes, which were not totally explained by adiposity [40]. Our study results also shows that leptin levels are significantly correlated with both TG/HDL-C ratio and HOMA-IR index, the biomarkers of IR.

Leptin affects autonomic function by acting both centrally and peripherally. Leptin receptors in hypothalamic brain regions, implicated in cardiovascular control, may exert a stimulatory effect on sympathetic activation, which results in autonomic hyperactivity due to direct effects of neuropeptide systems, such as the melanocortin and corticotropin-releasing hormone [41]. Additionally, leptin receptors are abundantly present in the carotid body (CB) [42]. CBs contain glomus, which are polymodal chemoreceptors, also known as peripheral chemoreceptors that detect multiple chemical changes in oxygen, carbon dioxide, PH, insulin, and blood sugar levels. A recent study showed that leptin increases the carotid sinus nerve activity [43], which transmits chemosensory input from the carotid bodies to the medullary centers (central chemoreceptors) in a mouse model. However, it is not yet clear how plasma leptin regulate autonomic function in patients with diabetes and prediabetes. Obesity triggers inflammation and oxidative stress, which activates the carotid bodies. The overactivation of the carotid bodies can contribute to increased sympathetic system activity and lead to hypertension and IR in T2DM [44].

### Risk factors associated with the severity of CAN

The pathophysiological mechanism of CAN development is multifactorial, and several studies have reported the important role of cardiovascular risk factors [7, 45–47]. CAN is a length-dependent pattern of disease, and parasympathetic activity can be damaged in the early phase of CAN with autonomic imbalance. As the disease progresses, sympathetic denervation occurs in the late stage of CAN [48]. Our results also shows that all parasympathetic parameters included in our study (e.g., HR_DB, VR, E:I ratio, 30:15 ratio) are significantly lower in patients with CAN than in those without CAN; however, the difference between sympathetic parameters (LF power, orthostatic BP change) is not obvious between groups. This finding is compatible with that of previous reports [2, 15, 16].

Regarding research on the relationship between leptin and CAN, one study from Japan shows that leptin is specifically associated with reduced HRV parameters in patients with DM compared with those without DM, with full adjustment of clinical parameters, which comprise quantitatively determined visceral adiposity [15]. Our results also show that leptin level is negatively correlated with all parasympathetic parameters included in our study (e.g., HR_DB, VR, E:I ratio, and 30/15 ratio). Another study from Korea shows a borderline significant positive correlation between leptin level and CAN score [16]. Autonomic dysfunction is seen early in the course of DM, and it occurs alongside alterations of various inflammatory adipocytokines [2]. Therefore, it highlights the concept of autonomic imbalance in the pathogenesis of CAN. With the progression of CAN, cardiovagal impairment is followed by sympathetic impairment. Increased leptin level could be secondary to autonomic imbalance or obesity-related leptin resistance [2].

Another important issue is whether the medications for modifying vascular risk factors (e.g., antihypertensive, antihyperlipidemic, and antihyperglycemic agents) can decrease leptin levels. One systematic review for meta-analysis of randomized placebo-controlled trials (RCTs) focuses on the impact of statin therapy on plasma leptin levels and shows that results are inconclusive [49]. Another meta-analysis of RCTs investigates the effects of pioglitazone on blood leptin levels in patients with T2DM and showed a significant difference, although relatively few RCTs are included, and a high level of statistical heterogeneity are found. Current studies have demonstrated that some antihypertensive medications may be more relevant than others in terms of reducing leptin levels in obesity. It seems that focus on pharmacologic suppression of the sympathetic nervous system, the renin–angiotensin system, and the blood pressure reduction effect is induced by weight loss. However, the results are also inconclusive [50]. Although our patients were well-controlled and almost all of them received medications to control the vascular risk factors, leptin levels remain significantly associated with the severity of CAN, according to both the CASS and CARTs score.

## Study Limitations

This study has several limitations. First, visceral adipose tissue is a bioactive organ that secretes several adipokines (e.g., leptin) and proatherogenic cytokines involved in cardiovascular events. A previous clinical study showed that visceral obesity revealed a more significant correlation with IR compared to central obesity, assessed by WC [51]. However, other quantitative approaches to assess visceral obesity, such as dual bioelectrical impedance analysis for visceral fat measurement [52], should be considered for future studies. Second, current evidence supports that aerobic exercise, alone or combined with hypocaloric diet, improves symptoms of MetS, and also alters systemic levels of adipokines [53]. Clinical studies conclude that intensified multifactorial intervention (hyperglycemia, hypertension, dyslipidemia, and microalbuminuria) reduced the risk of CAN progression [54] in T2D and T1D [55]. Although we observed a close relationship among WC, leptin level and severity of CAN in our observational study, a longitudinal study is needed to confirm that control of WC can decrease leptin levels and can be effective in reducing CAN progression.

## Conclusions

The coexistence of poor cardiovascular function, higher leptin level, and central obesity is demonstrated in patients with T2DM and prediabetes. It is been highlighted that these presentations are closely related to each other in this study. The results of mediation analysis provide possible pathophysiology for how both high WC and high leptin level adversely impact the severity of CAN. As the leptin level serves as mediator between central obesity and severity of CAN, a longitudinal study is needed to confirm that control of WC can decrease leptin levels and can be effective in reducing CAN progression.

## Data Availability

The data from this study can be acquired from the corresponding author upon reasonable request.

## Abbreviations

T1DM and T2DM: Type 1 and type 2 diabetes mellitus
CAN: Cardiovascular autonomic neuropathy
CASS: Composite autonomic scoring scale
CARTs: The cardiac autonomic reflex tests
TBARS: Thiobarbituric acid-reactive substance
HDL-C: High-density lipoprotein cholesterol
HOMA-IR: Homeostasis model assessment of insulin resistance
IR: Insulin resistance
MetS: Metabolic syndrome
HRV: Heart rate variability
BMI: Body mass index
SBP and DSP: Systolic and diastolic blood pressure
WC: Waist circumference
DR: Diabetic retinopathy
TG: Triglyceride
eGFR: Estimated glomerular filtration rate
HBA1c: Glycohemoglobin
hs-CRP: High-sensitive C-reactive protein
UACR: Urine albumin-creatine ratio
HR_DB: Heart rate response to deep breathing
VR: Valsalva ratio
HUT: Head-up tilt tests
CB: Carotid body
RCTs: Placebo-controlled trials
